# Imaging acute complications in cancer patients: what should be evaluated in the emergency setting?

**DOI:** 10.1186/1470-7330-14-18

**Published:** 2014-04-29

**Authors:** Marcos D Guimaraes, Almir GV Bitencourt, Edson Marchiori, Rubens Chojniak, Jefferson L Gross, Vikas Kundra

**Affiliations:** 1 Department of Imaging, AC Camargo Cancer Center, Rua Paulo Orozimbo, 726, Aclimação, Zip 01535-001 São Paulo, SP, Brazil; 2 Department of Radiology, Universidade Federal do Rio de Janeiro, Rua Thomaz Cameron, 438, Zip 25685-120 Valparaiso, RJ, Brazil; 3 Department of Thoracic Surgery, AC Camargo Cancer Center, Rua Antônio Prudente, 211, Liberdade, Zip 01509-010 São Paulo, SP, Brazil; 4 Department of Diagnostic Radiology, MD Anderson Cancer Center, The University of Texas MD Anderson Cancer Center, 1515 Holcombe Blvd, Zip 77030 Houston, TX, USA

## Abstract

Increased incidence world-wide of cancer and increased survival has also resulted in physicians seeing more complications in patients with cancer. In many cases, complications are the first manifestations of the disease. They may be insidious and develop over a period of months, or acute and manifest within minutes to days. Imaging examinations play an essential role in evaluating cancer and its complications. Plain radiography and ultrasonography (US) are generally performed initially in an urgent situation due to their wide availability, low cost, and minimal or no radiation exposure. However, depending on a patient’s symptoms, evaluation with cross-sectional imaging methods such as computed tomography (CT) and magnetic resonance imaging (MRI) is often necessary. In this review article, we discuss some of the most important acute noninfectious oncological complications for which imaging methods play an essential role in diagnosis.

## Introduction

Cancer has become one of the leading causes of natural deaths worldwide. The high incidence of neoplasms increased the medical care related to complications of this disease in recent years. These complications may present as an acute life-threatening or insidiously, taking weeks or months to be recognized and treated. Moreover, it is not uncommon such complications being the first manifestation of of the disease [1].

Cancer complications can be classified as the direct or indirect effects of a tumor. Direct or structural effects include invasion or mechanical compression of structures adjacent to the tumor. Indirect complications include systemic manifestations of the disease, such as hypercoagulability, immune suppression, and paraneoplastic syndrome. Surgical complications and those associated with the side effects of chemotherapy and/or radiation therapy are also significant [1,2]. Table 1 lists the more common acute oncological complications.

**Table 1:** Major noninfectious acute complications of cancer patients per system

**System**	**Complications**
**Neurologic**	Cord compression syndrome
	Intracranial hypertension/hydrocephalus
**Thoracic**	Superior vena cava syndrome
	Pericardial effusion/cardiac tamponade
	Pleural effusion
	Pulmonary thromboembolism
	Massive hemoptysis
**Abdominal**	Intenstinal obstruction
	Inflammatory intestinal changes
	Biliary obstruction
	Urinary tract obstruction
	Bleeding complications

Imaging examinations play an essential role in evaluating cancer and its complications. Plain radiography and ultrasonography (US) are generally performed initially in an urgent situation due to their wide availability, low cost, and minimal or no radiation exposure. However, depending on a patient’s symptoms, evaluation with cross-sectional imaging methods such as computed tomography (CT) and magnetic resonance imaging (MRI) is often necessary [3].

In this review article, we discuss some of the most important acute noninfectious oncological complications for which imaging methods play an essential role in diagnosis.

## Review

### Spinal cord compression syndrome

Spinal cord compression occurs in 2.5–6% of patients with cancer [4]. Early diagnosis of this oncological emergency is extremely important to prevent neurological sequelae such as paralysis and loss of bowel/bladder control, which may be permanent if diagnosis is delayed by even a few hours. The prognosis is poorer in the presence of paralysis or absence of a clinical response to treatment [1,2,5].

Most (80%) cases of spinal cord compression syndrome occur in patients with previous cancer diagnoses [6]. The most common and earliest symptom is back pain, present in 90% of patients, which can precede neurological symptoms by weeks [5]. Other symptoms include radicular pain, motor weakness, sensory deficiencies, gait disturbance, and urinary bladder or intestinal dysfunction. The main prognostic factor is the neurological state at the time of diagnosis; because long term neurological deficits may not respond to treatment, this diagnosis should always be suspected in patients with cancer who develop pain in the dorsal area [6-9].

The thoracic vertebral column is most commonly affected (70% of cases) [5]. The majority of cord compressions are of extradural origin, secondary to metastatic vertebral lesions that cause cortical erosion and impress upon into the spinal canal. Cancers of the breast, lung, and prostate are most frequently associated with this condition, accounting for nearly two-thirds of all cases [4]. Less frequently, tumors involving the paravertebral area, such as lymphomas, sarcomas, and lung cancer, can invade through intervertebral foramina and impress upon the spinal cord. More common non-neoplastic causes of cord compression that may be seen in the setting of cancer include spinal fractures and abscesses [6-8].

MRI is the gold standard for the diagnosis of cord compression [5,6]. This imaging modality enables definition of the extent of the compressed area and aids treatment planning, such as radiation therapy. The use of paramagnetic intravenous contrast improves the method’s sensitivity, including in identifying leptomeningeal or intramedullary metastases.

**Figure 1:**
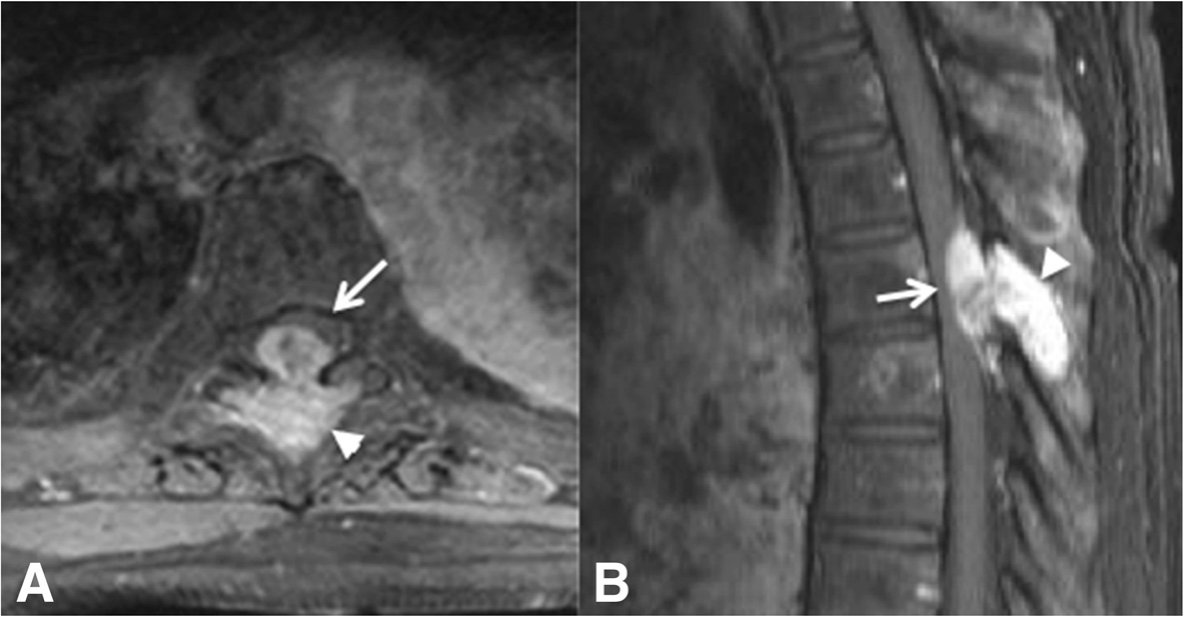
**A 61-year-old woman with breast cancer metastatic to the spine leading to spinal cord compression syndrome.** Axial **(A)** and sagittal **(B)** post-contrast T1-weighted MR images of a patient with metastatic breast cancer showing a bone lesion in the posterior elements of T6 (arrowhead), with high T2 signal intensity and intense contrast enhancement, impressing upon the spinal canal and dislocating the cord anterolaterally (arrow).

On MRI, malignant tumors in the spinal column generally present with low signal intensity on T1-weighted images, high signal intensity on T2-weighted images, and contrast enhancement. Such tumors impress upon the spinal canal, dislocating and compressing the spinal cord. This can result in high signal intensity of the cord on T2-weighted images, which suggests edema (Figure 1).

When MRI is not available or is contraindicated, CT with myelography is the method of choice [4]. When this is not available, CT preferably with intravenous contrast may be performed. Bone scintigraphy and plain films can show bone alterations, but do not visualize the cord.

### Intracranial hypertension

Increased intracranial pressure is a common and potentially serious neurological complication in patients with cancer [4,10-12]. It is caused mainly by intraparenchymal metastatic disease. Among malignant tumors, lung cancer, breast cancer, and melanoma most commonly spread to the brain [4]. Other causes of increased intracranial pressure include intratumor hemorrhage and hydrocephalus. Patients with cerebral metastases of melanoma, choriocarcinoma, and renal cell carcinoma are at higher risk for bleeding. Hydrocephalus is most commonly obstructive or non-communicating, caused by lesions at the level of the foramen of Monro, the aqueduct of Sylvius, or the fourth ventricle, but can also be non-obstructive or communicating in patients with diffuse leptomeningeal carcinomatosis, which obstructs the arachnoid granulations, impeding cerebrospinal fluid reabsorption [10-12].

Elevated intracranial pressure can result in general symptoms, such as headache, nausea, vomiting, and reduced level of consciousness [11]. Headache is present in about half of all patients with (primary or secondary) cerebral tumors, especially those showing rapid or infratentorial growth. Projectile vomiting without nausea is frequently observed in patients with tumors of the posterior fossa, which evolve into obstructive hydrocephalus [11]. Other symptoms related to intracranial hypertension secondary to neoplastic disease are focal neurological dysfunction, cognitive deficits, and convulsions. Elevated intracranial pressure and the effect of the mass can cause ischemic encephalic vascular trauma and brain herniation [10-12].

**Figure 2:**
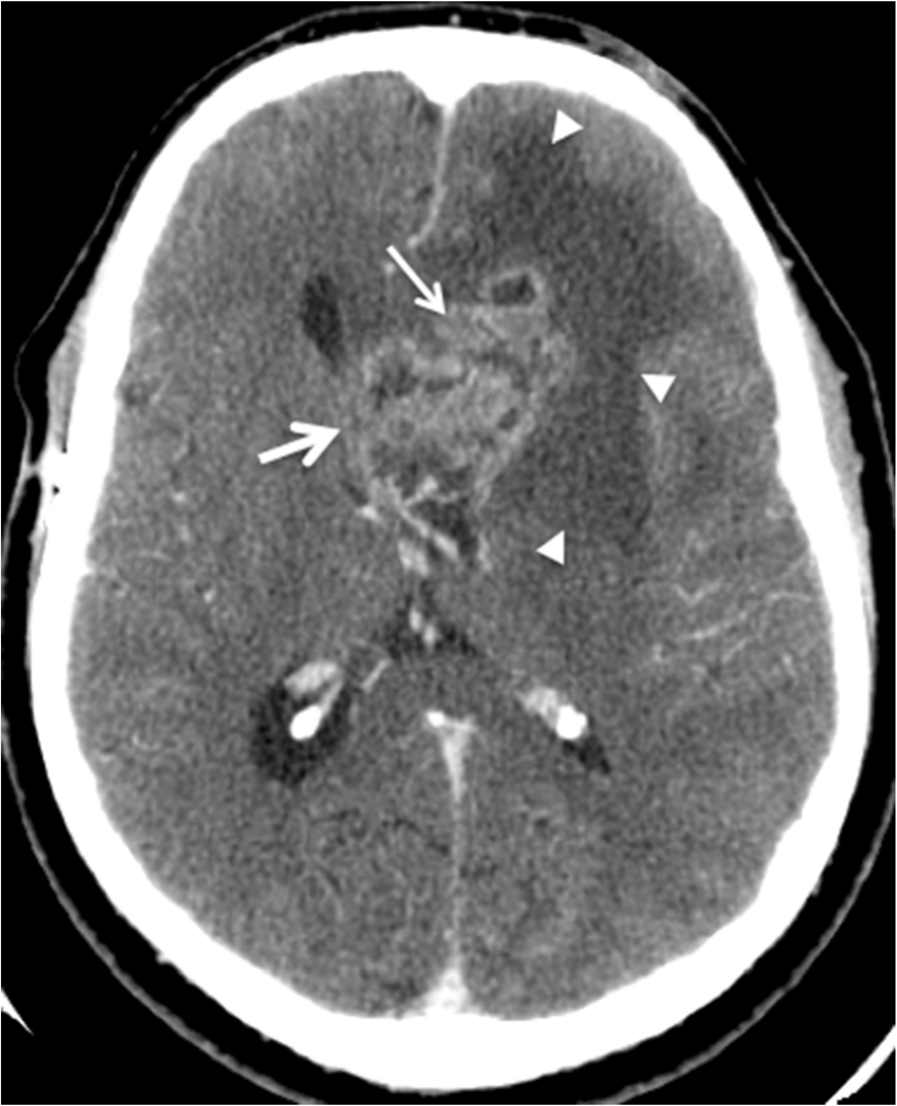
**A 37-year-old man with intracranial tumor with mass effect.** Axial post-intravenous contrast images. Computed tomographic images of a patient with glioblastoma multiforme in the left frontal lobe. The lesion with irregular contours and heterogeneous enhancement (thin arrow) has mass effect characterized by hypoattenuation (edema) of the adjacent white matter (arrowheads), compression of the lateral ventricle, and contralateral deviation of the medial line structures, with signs of subfalcine herniation (thick arrow).

Although MRI is best for evaluating the central nervous system, CT is generally the first examination performed in urgent cases of intracranial hypertension [4]. On CT, one can see mass effect (Figure 2), acute hemorrhage as increased attenuation on non-contrast-enhanced CT, hydrocephalus and herniation [12]. MRI is more sensitive for the identification of small metastases and leptomeningeal implants, and can serve as a complementary imaging modality when CT shows no remarkable abnormality [1,4].

### Superior vena cava syndrome

Superior vena cava syndrome results from partial or complete obstruction of the blood flow in the superior vena cava, causing reduction in venous return to the head, neck, and upper limbs [1,2,13]. Although it is considered a classic oncological emergency, it is rarely immediately life threatening [1].

Symptoms include cough, dyspnea, dysphagia, edema, and congestion in the neck, face, and upper limbs. Collateral venous circulation can cause distension of the surface veins of the thoracic cavity wall [14-17].

More than 50% of patients with superior vena cava syndrome become symptomatic after receiving a diagnosis of cancer due to severe clinical worsening of these patients [1,17,18]. The prognosis for this syndrome depends on that for the underlying disease. Malignant tumors such as lung cancer, lymphomas, and metastatic mediastinal tumors are responsible for more than 90% of cases [15-17]. Venous thrombosis combined with the presence of a catheter within the superior vena cava is an uncommon cause of this condition in patients with cancer.

**Figure 3:**
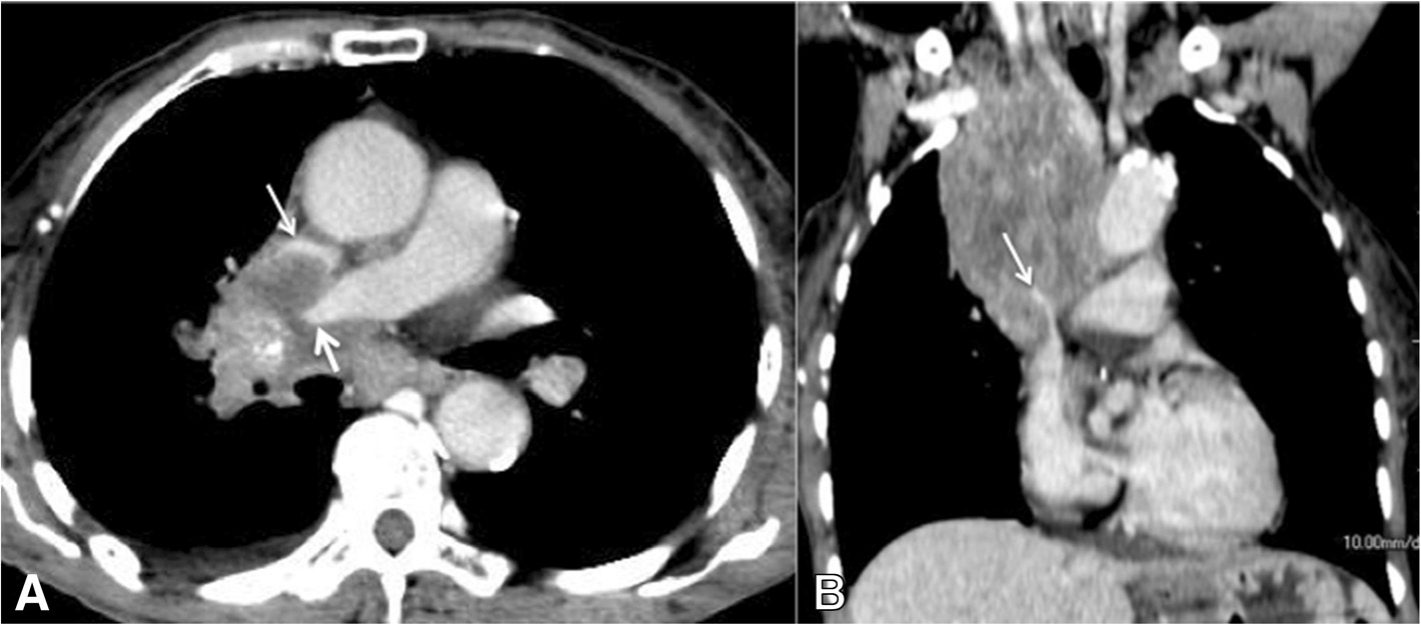
**A 55-year-old man with superior vena cava syndrome.** Computed tomographic images of a patient with metastatic laryngeal cancer showing an infiltrative mediastinal mass causing compression of the right pulmonary artery (thick arrow) and superior vena cava (thin arrow). **(A)** Post-contrast axial slice. **(B)** Post-contrast coronal reconstruction.

Although superior vena cava syndrome is diagnosed clinically, CT is generally used to confirm it through visualization of superior vena cava luminal narrowing in association with the tumor mass (Figure 3) or thrombosis [19]. CT can reveal the site of obstruction and enables the differentiation of extrinsic compression caused by tumor versus intravascular thrombosis. CT can also provide additional information about the tumor, such as its size and its relationship to other mediastinal structures [17,18].

CT should be performed with intravenous contrast and images acquired in the later phases to guarantee optimal contrast of the brachiocephalic veins and to avoid streak artifacts from arterial contrast [14,17]. If iodinated contrast cannot be used MRI may also be performed. Sequences with and without contrast as well multiplanar reconstruction aide MRI evaluation of tumor extent and the compromise of the superior vena cava and adjacent anatomical structures. Cross-sectional imaging is beneficial for therapeutic planning, especially in patients with conditions requiring a surgical approach [14-18].

### Pericardial effusion with cardiac tamponade

Malignant pericardial effusions are present in 10–15% of patients with cancer and are caused by the obstruction of lymphatic drainage, direct extension or hematogenous metastasis [18,19]. Pericardial effusion is generally a late finding in patients with metastatic cancer. The most common causes are lung and breast cancers, followed by melanoma, leukemia, and lymphoma [1,2,19]. Benign inflammatory pericardial thickening and effusion may arise as side effects of radiation therapy and certain chemotherapies or from infectious causes in immunocompromised patients. Two-thirds of patients with this condition are asymptomatic. The most common symptoms are dyspnea, orthopnea, fatigue, palpitations, and dizziness [19].

Cardiac tamponade occurs when a quantity of liquid that has accumulated in the pericardial sac causes restriction in diastolic expansion and hemodynamic instability [20]. This is more common with rapid fluid accumulations rather than slow accumulation. The main signs on physical examination are paradoxical pulse, tachycardia, hypotension, distension of the cervical veins, weak peripheral pulse, and muffled heart sounds [20].

Echocardiography is the main modality used to confirm a diagnosis of pericardial effusion, to evaluate its hemodynamic impact, and to guide pericardiocentesis [21]. Cytological examination of the pericardial fluid should be performed to confirm or exclude the presence of neoplastic cells.

**Figure 4:**
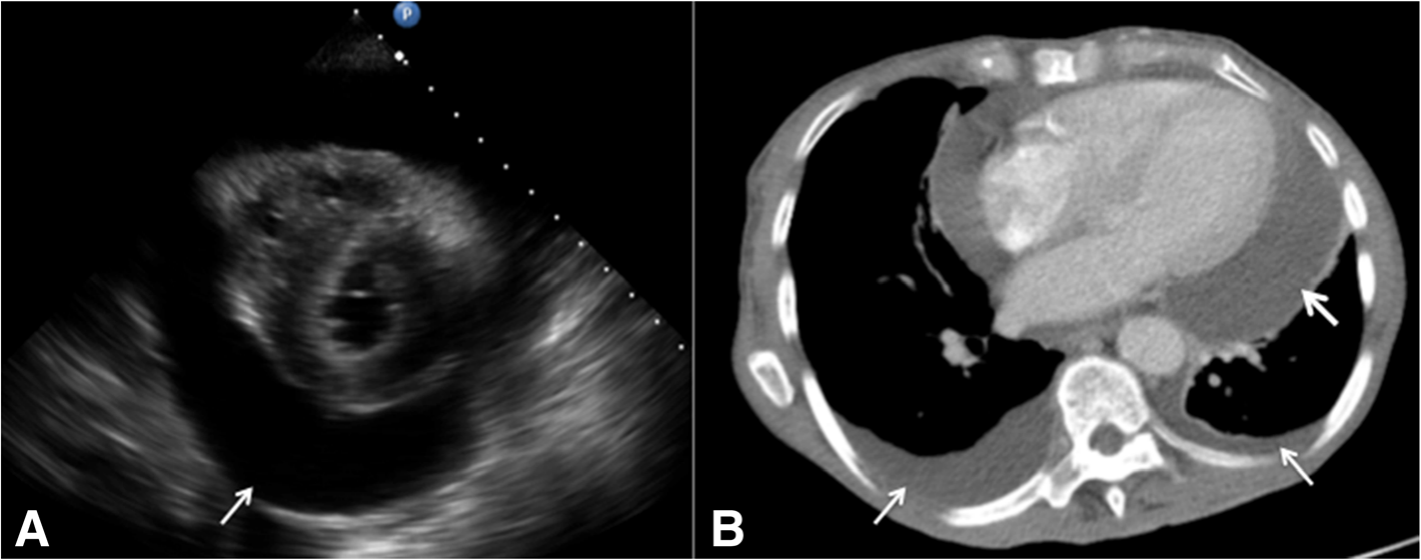
**A 72-year-old man with history of lung cancer presenting pericardial effusion/cardiac tamponade. (A)** Echocardiogram demonstrating hypoechoic pericardiac fluid (arrow) and through transmission. **(B)** Axial computed tomographic slice of the thorax obtained in a mediastinal window after intravenous contrast administration showing a large pericardial effusion (thick arrow) and small bilateral pleural effusions (thin arrows).

In some cases, radiography and thoracic CT performed for other clinical reasons show signs of pericardial effusion [19-21]. Chest x-rays may show an increased transverse diameter of the cardiac area. CT may show pericardial thickening with fluid attenuation in most cases (Figure 4), but greater density may be due to the presence of debris or hemorrhage. Evidence of irregular thickening or tumor nodules in the pericardium is rare. CT can also reveal signs of direct cardiac insufficiency, such as hepatic congestion seen as patchy attenuation of the liver and reflux of the contrast medium into the inferior vena cava and hepatic veins [9,18,19].

### Pleural effusion

Benign and malignant pleural effusions are common in patients with cancer. These can lead to compression of the adjacent pulmonary parenchyma and when large, difficulty breathing. Patients are commonly asymptomatic, but may present with dyspnea, cough, thoracic pain, weight loss, anorexia, and/or fatigue [22].

Benign pleural effusions may be secondary to compromised lymph drainage, to infectious/inflammatory processes, or to reduced oncotic pressure. Malignant pleural effusions are typically caused by pleural compromise from the underlying disease. Malignancies that most frequently affect the pleura are lung, breast, and ovarian cancers and lymphoma. Primary pleural tumors, such as mesothelioma are quite rare and generally cause effusion associated with the pleural mass [23,24].

**Figure 5:**
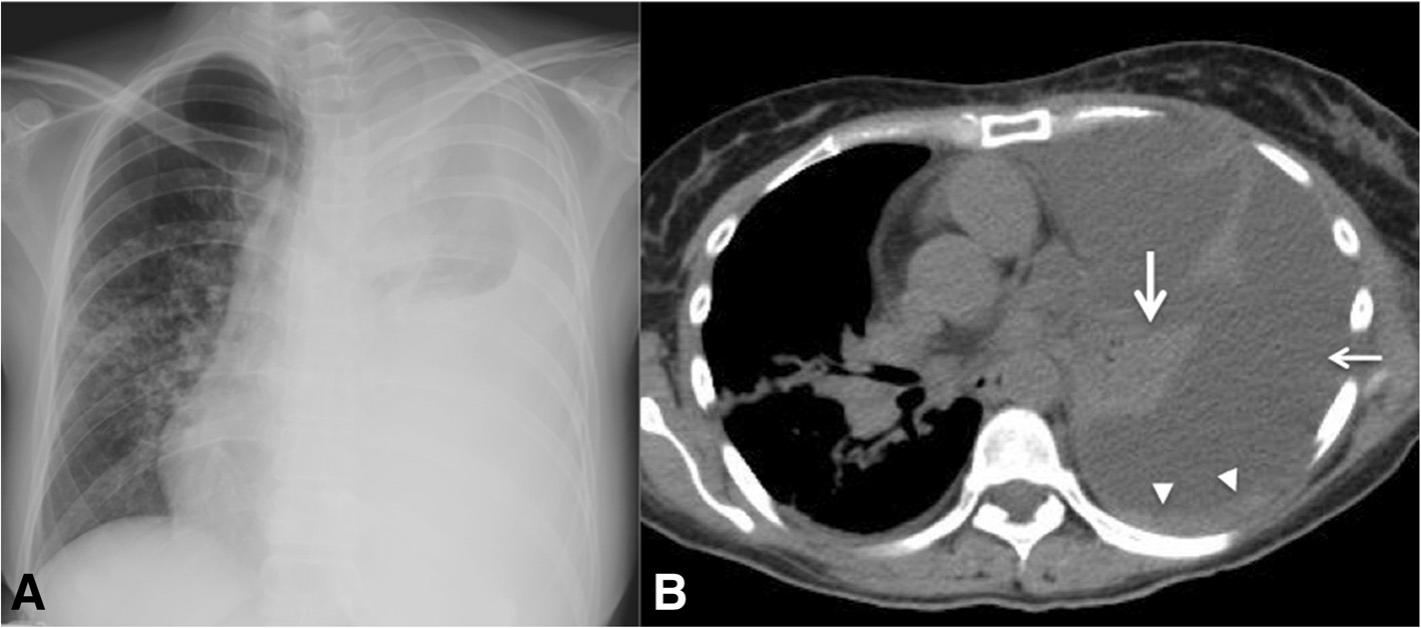
**A 36-year-old woman with breast cancer presenting severe pleural effusion in the left hemithorax. (A)** Anteroposterior radiograph of the thorax showing opacification of the left hemithorax and slight contralateral deviation of the mediastinal structures. **(B)** Axial computed tomographic image of the thorax obtained in the mediastinal window without contrast showing a large left pleural effusion (thin arrow), pleural thickening (arrowhead), and collapse of the left lower lobe (thick arrow).

Suspected pleural effusion can be confirmed by radiography or ultrasound; the latter modality is also useful in guiding thoracocentesis [24,25]. CT is important for the evaluation of pulmonary parenchyma, exclusion of other causes of dyspnea, and assessment of signs of malignancy. Although most malignant pleural effusions have a simple appearance on CT, with fluid attenuation and without pleural thickening, the presence of circumferential or nodular pleural thickening suggests malignancy (Figure 5) [26]. Positron emission tomography/CT can be useful in identifying the most suspicious areas of pleural thickening to guide percutaneous needle biopsy [22-25].

### Pulmonary thromboembolism

Deep vein thrombosis and pulmonary thromboembolism (PTE) are common complications in patients with cancer because of their hypercoagulable state, local tumor effects, or treatment side effects [27]. Malignant tumors most frequently associated with the development of PTE are lung, colon, and prostate cancers [28]. The incidence of venous thromboembolism is higher in patients receiving chemotherapy, reaching 10% in patients with ovarian cancer or lymphoma and 28% in patients with malignant gliomas [29]. Patients with cancer and PTE also have a poorer prognosis, with mortality rate four to eight times that of the general population with PTE [27-30].

It is found incidentally on imaging examinations performed for other reasons in about 4% of patients with cancer [31] and mainly affects small pulmonary arteries. This finding should be reported urgently and treatment initiated because it is associated with the presence of deep vein thrombosis and the development of new thromboembolic events.

PTE is commonly asymptomatic or associated with non-specific symptoms. Acute onset dyspnea is the most suggestive symptom of PTE, followed by pleuritic pain. Massive PTE can cause pulmonary hypertension and signs of direct cardiac insufficiency. Chemotherapy, history of recent surgery, prolonged immobilization, or signs of deep vein thrombosis are associated with developing PTE [27-29].

The differential diagnosis of PTE includes pulmonary tumor thrombotic microangiopathy (PTTM), an extremely rare and serious complication in patients with cancer caused by the presence of microemboli; it is associated with adenocarcinomas, mainly of gastric origin [32]. A patient with PTTM develops rapidly progressing signs and symptoms of pulmonary hypertension and cardiac failure, evolving to death in a few days [33,34]. Diagnosing this condition is extremely difficult, and in many cases is performed post-mortem.

CT angiography is the method of choice to diagnose and evaluate the extent of PTE. CT should be performed using a specific angiographic protocol to achieve adequate contrast of the pulmonary arteries, with suitable venous access. Multiplanar reconstructions can be useful in identifying the thrombus [32-35].

**Figure 6:**
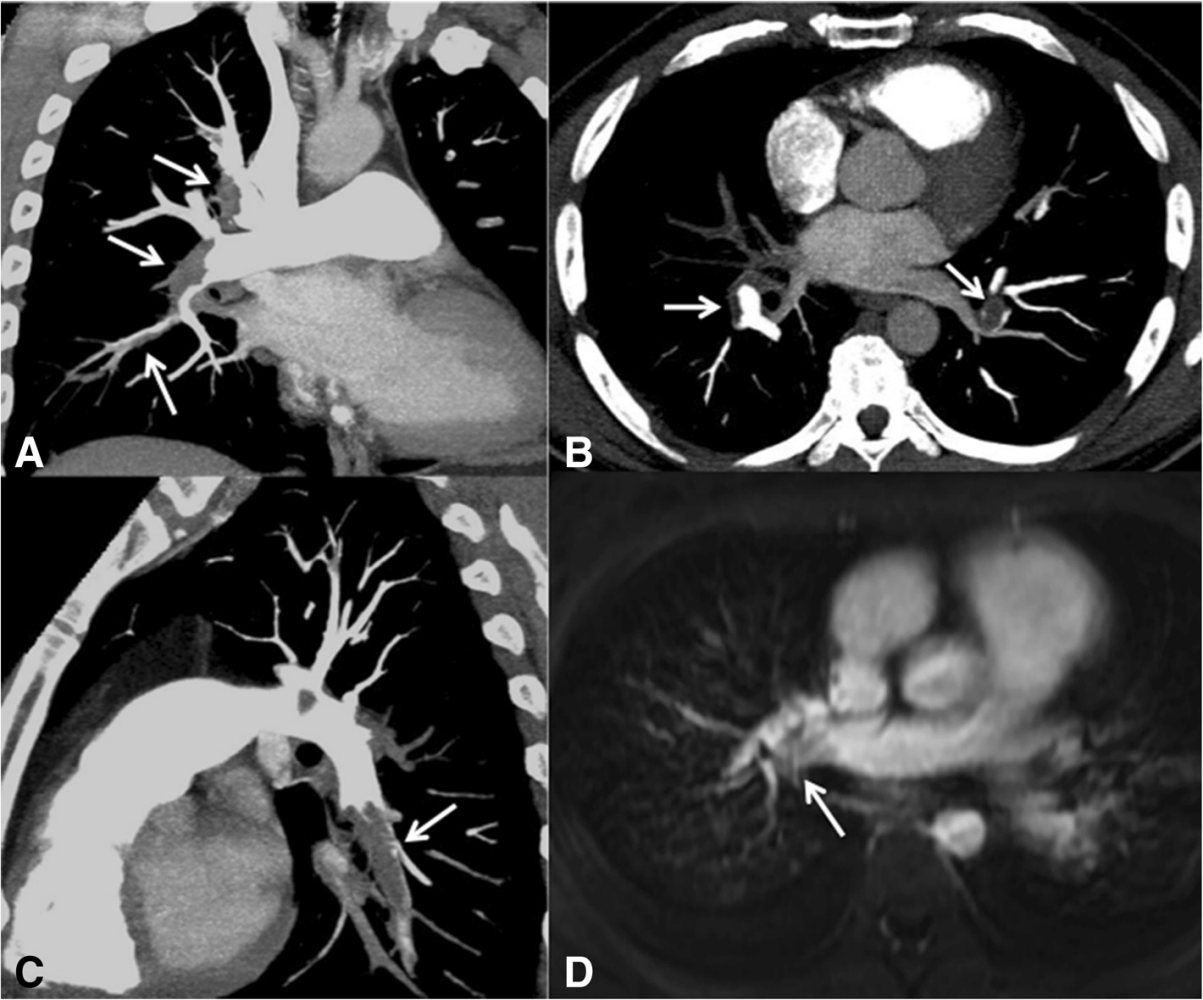
**A 32-year-old woman with lymphoma presenting severe acute pulmonary thromboembolism.** CT and MR images of the pulmonary arteries demonstrating pulmonary emboli. **(A)** CT, coronal maximum intensity projection (MIP) reconstruction demonstrating filling defects at the bifurcation and upper and lower segments of the right pulmonary artery (white arrows). **(B)** CT, Axial slice demonstrating filling defects at the bifurcation of the right and left pulmonary arteries (white arrows). **(C)** CT, sagittal reconstructions demonstrating filling defects in the inferior lobar branch of the left pulmonary artery (white arrow). **(D)** MRI with balanced steady state free precession sequence demonstrating filling defects in the right pulmonary artery (white arrow).

Acute PTEs are identified as one or more filling defects in a pulmonary arterial branch (Figure 6). Some cases include signs of pulmonary infarction, characterized by a wedge-shaped peripheral pulmonary parenchymal opacity with a pleural base and an apex oriented toward the occluded pulmonary artery. CT also provides the opportunity to diagnose alternative conditions, such as pneumonias or other pulmonary parenchymal diseases, or pericardial, pleural, or mediastinal disease that can contribute to dyspnea or thoracic pain.

In patients who cannot undergo CT angiography, the method of choice is ventilation/perfusion pulmonary scintigraphy. However, this modality may be unavailable in urgent situations in many institutions. Another option is MRI of the thorax, which enables the identification of large thrombi with a balanced steady state free precession sequence, without the use of paramagnetic contrast (Figure 6) [36]. Thoracic x-rays may be normal or yield nonspecific findings, such as focal opacities and a small pleural effusion. Nonspecific and uncommon x-ray signs have been described in patients with PTE, including Hampton’s hump (peripheral triangular opacity with a pleural base), the Fleischner sign (enlargement of the pulmonary artery on the side of the PTE), and the Westermark sign (pulmonary oligemia distal to the PTE) [18].

PTTM shows nonspecific signs of pulmonary hypertension on thoracic CT images. Because of changes to the small vessels, typical findings of infectious bronchiolitis, such as diffuse centrilobular opacities and a tree-in-bud pattern, have also been described [32-35].

### Intestinal obstruction

Intestinal obstructions are relatively common in patients with cancer and can be caused by benign etiologies or directly associated with the tumor [37,38]. Benign causes include postoperative adhesions, actinic sequelae, and inflammatory and infectious changes. Malignant causes include obstruction by the primary tumor, recurrence, and metastasis [38]. The clinical manifestations of benign and malignant obstructions are very similar, and imaging findings may be inconclusive. However, differential diagnosis has important implications for prognosis and treatment [39,40].

Malignant causes of small intestinal obstruction are less frequent than adhesions and inflammatory changes. Malignant obstruction of the small intestine is caused more frequently by metastatic tumors than by primary tumors of the small intestine, which are responsible for <2% of all gastrointestinal neoplasias [40]. Malignant obstruction of the colon is generally caused by primary colorectal carcinoma [41,42].

Focal intestinal lesions can also cause intussusception [43,44]. This condition is generally rare in adults, responsible for only 5% of intestinal obstructions [44,45]. In addition to foreign bodies, primary neoplasias of the small intestine and colon and metastatic lesions (e.g., melanoma) can cause intussusception. When a neoplasia is suspected, care should be taken to differentiate a real tumor mass from a pseudomass caused by intussusception [43-46].

Radiography can show signs of intestinal obstruction, with gaseous distension of the small bowel or bowel loops forming air-fluid levels on upright images [44]. Small bowel follow-through is not done very commonly presently. CT is generally performed to evaluate the site and possible cause of obstruction and treatment planning. CT findings that suggest malignant intestinal obstruction are the presence of irregular parietal thickening or a mass with soft-tissue density at the point of transition [42-44]. More commonly, no mass is found and the obstruction is due to adhesions.

**Figure 7:**
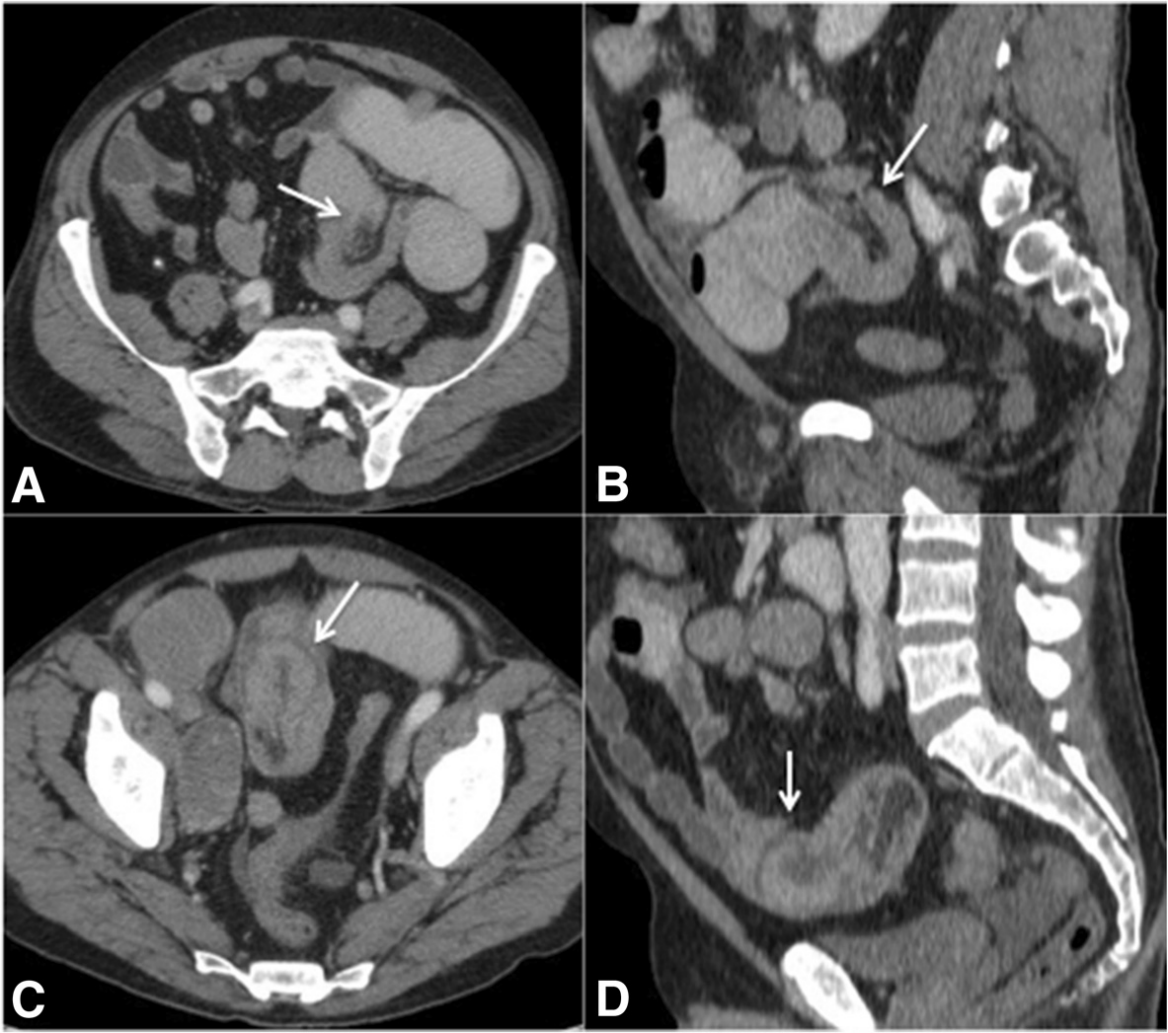
**A 29-year-old man Intestinal obstruction caused by intussusception.** Patient with metastatic melanoma evolving into a clinical picture of intestinal obstruction. Computed tomographic images obtained after oral and intravenous iodinated contrast show intussusception with distention of the upstream small bowel loops. **(A)** Axial and **(B)** sagittal images showing the proximal portion of intussusception and invagination of the mesenteric adipose tissue toward its interior (arrows). **(C)** Axial and **(D)** sagittal images showing the distal portion of intussusception with parietal thickening of the invaginated small intestinal loops (arrows), which was confirmed as a melanoma metastasis after surgical resection.

In intussusception, CT may shows pathognomonic changes, such as the target sign or a “loop within loop” pattern, with or without invaginated fat and mesenteric vessels (Figure 7).

### Inflammatory intestinal changes

Acute intestinal inflammatory changes are common in patients with cancer, and various etiologies may be involved in these processes [46-48]. Neutropenic colitis or typhlitis is a cancer emergency that demonstrates transmural inflammation of the cecum, proximal colon, and terminal ileum [49]. It can develop in immunocompromised children and adults, for example those undergoing treatment for leukemia, receiving chemotherapy, or that have undergone bone marrow transplant. Early identification of this condition is fundamental because it can evolve into intestinal necrosis and has high morbidity and mortality rates. Patients normally present with fever, neutropenia, and abdominal pain [50].

Pseudomembranous colitis is caused by *Clostridium difficile* infection, commonly after antibiotic treatment, it should be considered especially in immunocompromised cancer patients with abdominal complaints [51,52]. It is the most common cause of diarrhea in hospitalized patients. Immunosuppressed patients undergoing chemotherapy who use broad-spectrum antibiotics are at risk of developing this complication. Diarrhea, abdominal pain, and fever typically manifest 1 week after the initiation of antibiotic therapy. In some cases, this condition can evolve into diffuse colitis and toxic megacolon [53].

Malignant tumors can also be associated with mesenteric ischemia and ischemic colitis [53]. Ischemia can be secondary to vascular occlusion caused by tumor compression/invasion or bacterial proliferation associated with intestinal distension and chronic stasis [53].

The small intestine and colon are sensitive to radiation therapy. Thus, actinic enteritis and colitis should be considered in the differential diagnosis when signs of an inflammatory process are present in previously irradiated areas [54].

Ultrasound is useful in some cases, but CT is generally the best method for the evaluation of intestinal complaints [37,40,46]. CT is also useful for excluding other causes of abdominal pain, including obstructions and inflammatory changes not associated with cancer, such as appendicitis, diverticulitis, and inflammatory bowel diseases [39,40,50].

**Figure 8:**
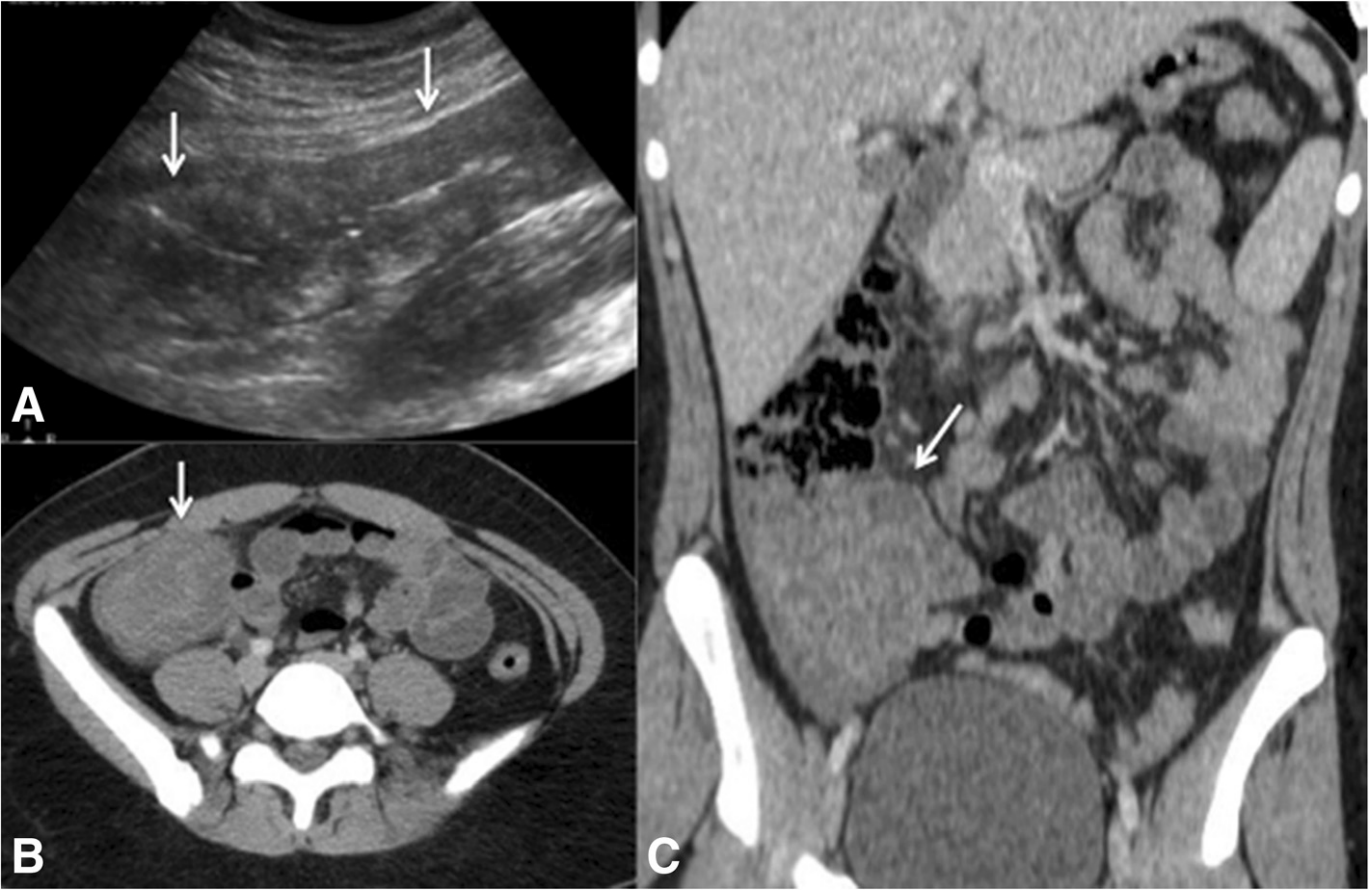
**A 16-year-old boy with neutropenic typhlitis.** Patient under treatment for leukemia, presented with abdominal pain in the right iliac fossa. **(A)** Ultrasound image showing diffuse thickening of the walls of the cecum and ascending colon (arrows). **(B)** Axial and **(C)** coronal computed tomographic slices obtained after intravenous contrast administration confirmed the ultrasound findings, showing thickening of the cecum wall and proximal portion of the ascending colon (arrows), compatible with typhlitis.

CT may show nonspecific inflammatory findings, including thickening and of the small intestine or colon, with or without effacement and striations of pericolic adipose tissue. The identification of pneumatosis, pneumoperitoneum, and pericolic collections often suggest developing necrosis or perforation requiring urgent surgical evaluation [40,50]. The presence of these findings in the cecum, the proximal portion of the ascending colon, and/or the distal ileum in an immunocompromised patient suggests a diagnosis of typhlitis (Figure 8) [49]. Pseudomembranous colitis is characterized by diffuse wall thickening generally along the entire colon, described as an “accordion sign” [51,52].

### Biliary obstruction

Biliary obstruction can be secondary to biliary stasis in patients with diffuse metastatic infiltration of the liver, causing obstruction of the small intrahepatic bile ducts, or it can occur due to compression of the main bile ducts in patients due to more commonly metastatic disease or lymphoma or disease within the ducts, for example, from cholangiocarcinoma [55].

Malignant tumors of the head of the pancreas and the ampulla of Vater are common causes of obstruction of the main bile ducts. Benign differential considerations include pseudocyst from pancreatitits [56].

Bile duct obstruction manifests mainly as jaundice, dark urine and pale-colored stools. In a patient with abdominal pain and/or fever, the possibility of cholangitis associated with biliary obstruction should also be considered [55,56].

Imaging methods should be able to define the presence or absence, level, and cause of bile duct obstruction. Ultrasound is generally the first method used and has good diagnostic accuracy in detecting dilation of the intra- and extrahepatic bile ducts [57]. A literature review showed that ultrasound has a sensitivity of 71% in delineating the level of obstruction and 51% in defining the etiology. Fat and gastrointestinal gas may limit evaluation [55].

Other methods for evaluating the bile ducts include endoscopic retrograde cholangiopancreatography (ERCP), which also allows biopsy. However, this is invasive, more complex and commonly less readily available than CT or MRI. Commonly, the second line test due to ease of availability is CT. MR also visualizes visceral structures, and one can employ heavily T2-weighted MR sequences for visualizing the ducts (MRCP) [56].

In cases of diffuse metastatic infiltration of the liver, CT or MRI shows multiple hepatic lesions that are more commonly infiltrative or confluent depending on the primary tumor, together with focal dilatation of the small intrahepatic bile ducts adjacent to vessels of the hepatic triad [55].

**Figure 9:**
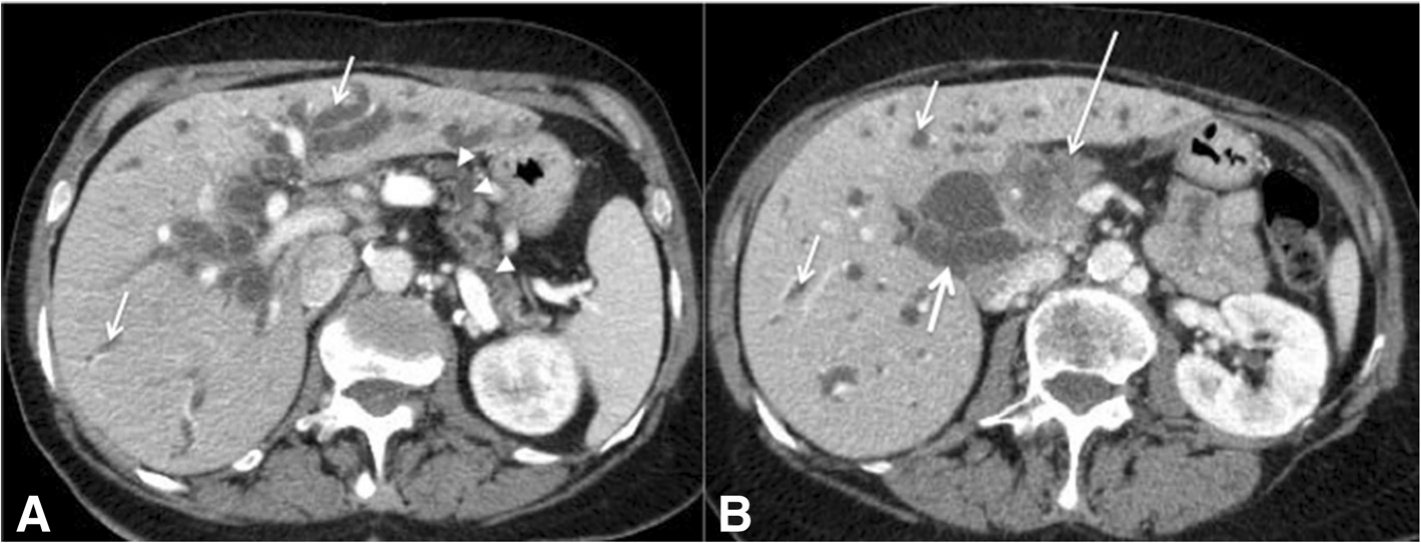
**A 58-year-old man with biliary obstruction caused by adenocarcinoma of the pancreas.** Axial **(A)** and **(B)** CT images of the abdomen obtained after intravenous contrast administration demonstrating dilatation of the intra- (thin arrows) and extrahepatic bile ducts (thick arrow), a pancreatic head mass (adenocarcinoma) with irregular contours and poorly defined borders (long arrow), and dilatation of the pancreatic duct (arrowheads).

In cases of main bile duct compression, diffuse dilation of the intra- and extrahepatic bile ducts is present upstream of the obstruction site (Figure 9). Such tumors are commonly in the porta-hepatis, pancreas or at the ampula. With pancreatic or ampullary tumors, one often sees a “double duct sign” with dilatation of both the biliary system and pancreatic duct. However, a normal-caliber main pancreatic duct does not exclude the diagnosis. Cholangiocarcinomas can have different presentations ranging from an intraluminal polypoid lesion of a bile duct, wall thickening or an infiltrative mass commonly with ill-defined borders [55,56,58].

### Urinary tract obstruction

Urinary tract obstruction can occur in patients with retroperitoneal or pelvic tumors; these are more commonly gynecological or urological cancers of the cervix, ovaries, bladder, and prostate [1,57]. Metastatic disease, for example, from gastric cancer can also be seen. Sarcoma or lymphoma as a cause is relatively rare. Unilateral urinary obstruction does not normally cause acute renal dysfunction because of compensation by the contralateral kidney. Urinary obstruction can be seen post surgery due to fibrosis involving the ureters. Obstruction of the urinary tract should be suspected in patients with complaints of pain in the flank and sudden anuria who have increased serum creatinine levels [57,59].

**Figure 10:**
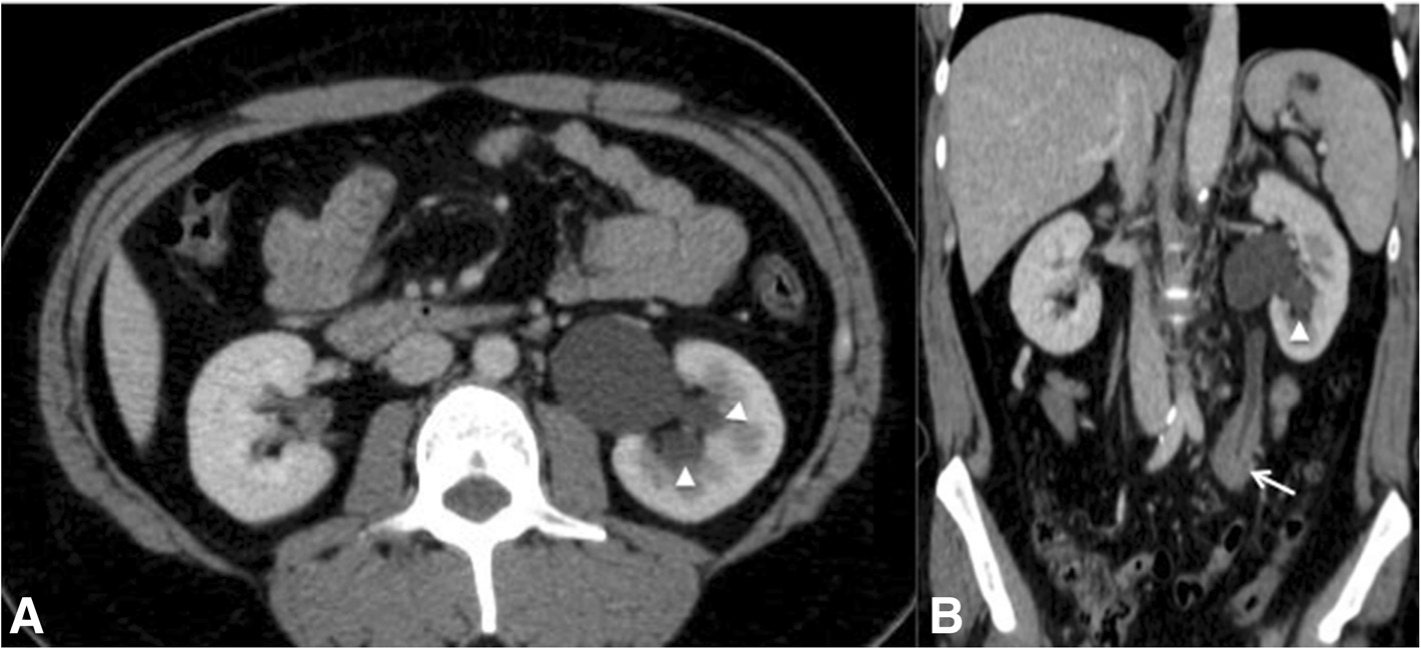
**A 23-year-old man with urinary obstruction. (A)** Axial and **(B)** coronal CT images of the abdomen obtained after intravenous contrast administration showing mild hydronephrosis (arrowhead) on the left, with a discrete delay in the concentration of intravenous contrast by the left kidney caused by a soft-tissue density mass (lymphoma) involving the middle third of the left ureter (arrow).

Ultrasound is the easiest way to detect the presence of hydronephrosis [57,59]. However, CT can be superior for determining the precise location of the obstruction, particularly when a pelvic or retroperitoneal mass is present. Unilateral obstruction is usually characterized by focal lesions in the urinary tract with soft-tissue density and upstream urethral dilation (Figure 10) [57,59]. In bilateral obstruction, the most common imaging findings are a large heterogeneous mass involving both ureters and causing bilateral ureterohydronephrosis. Intravenous iodinated contrast should be used with caution because patients can already have a degree of renal dysfunction, which may be exacerbated upon exposure to intravenous contrast. MR urography is an effective alternative; it enables identification of the site and cause of the obstruction in the majority of cases [60]. A heavily T2-weighted sequence may be used, but single shot T2 - fast spin echo (FSE) and balanced steady state free precession sequences can also serve to identify hydronephrosis and a mass [60].

### Bleeding complications

Cancer patients can develop bleeding complications secondary to thrombocytopenia, tumor rupture, or hemorrhage from a vascular neoplasm.

Massive hemoptysis is generally used to describe the expectoration of a large amount of blood and/or a rapid rate of bleeding, and when it occurs secondary to malignant disease, the mortality rate may be as high as 60%. Bronchogenic carcinoma is the most common cause of massive hemoptysis in patients older than 40 years. Endobronchial metastases from carcinoid tumors, breast, colon or kidney cancer, melanoma and sarcomas may also cause hempoptysis. Hemoptysis in cancer patients may also be caused by nonmalignant conditions, such as fungal infections, or may be related to thrombocytopenia or other coagulation disorders [1,61].

**Figure 11:**
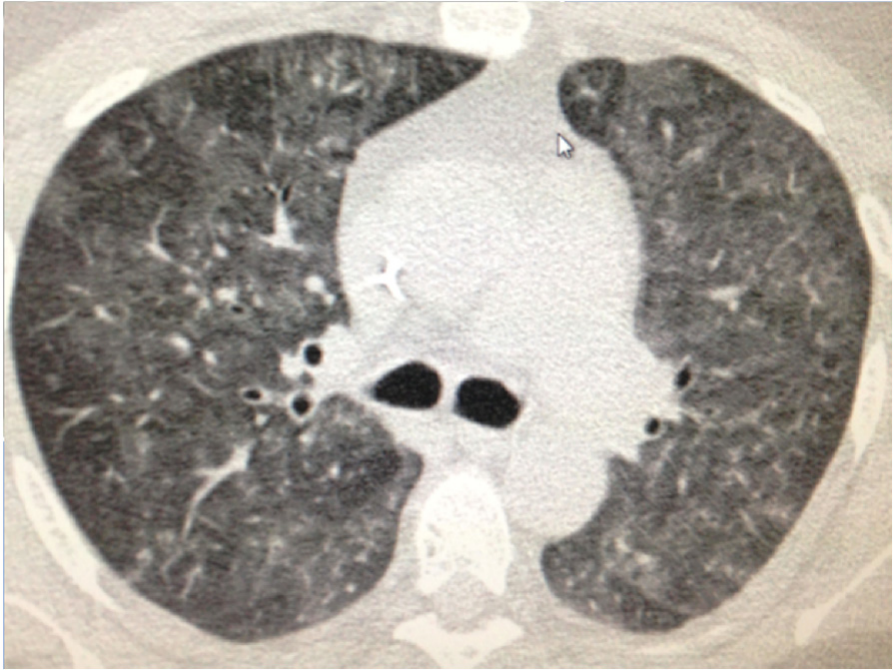
**A 24-year-old man on chemotherapy for non-Hodgkin lymphoma presenting with thrombocytopenia and hemoptysis.** Axial unenhanced CT image of the thorax showing diffuse bilateral ground-glass opacities, compatible with alveolar hemorrhage.

In patients with cancer-related massive hemoptysis, chest radiography may demonstrate abnormalities such as tumors, cavitary lesions, pulmonary infiltrates (Figure 11), and mediastinal masses. In stable patients, multidetector CT of the chest can be performed before bronchoscopy to help identify the site, cause, and vascular source of bleeding. Multidetector CT angiography play a crucial role in assessing the origin and course of the abnormal bronchial arteries and nonbronchial systemic collateral vessels responsible for hemoptysis, directing the interventional radiologist prior to catheter angiography [61,62].

Severe abdominal bleeding in cancer patients is a rare, but potentially fatal complication that requires prompt diagnosis and treatment. Malignant hemorrhage can occur in an organ parenchyma or subcapsular space due to direct tumor rupture or in the peritoneal cavity from carcinomatosis or extension of visceral tumor rupture. Hypervascular tumors, such as hepatocellular carcinoma, renal cell carcinoma, and melanoma, are the most commonly associated to spontaneous hemoperitoneum. Large size of the mass, a peripheral or subcapsular location and increased vascularity are the most important risk factors for intratumoral hemorrhage and subsequent spontaneous rupture. In addition, patients with hematologic malignancies can develop spontaneous hepatic or splenic rupture [40,61].

**Figure 12:**
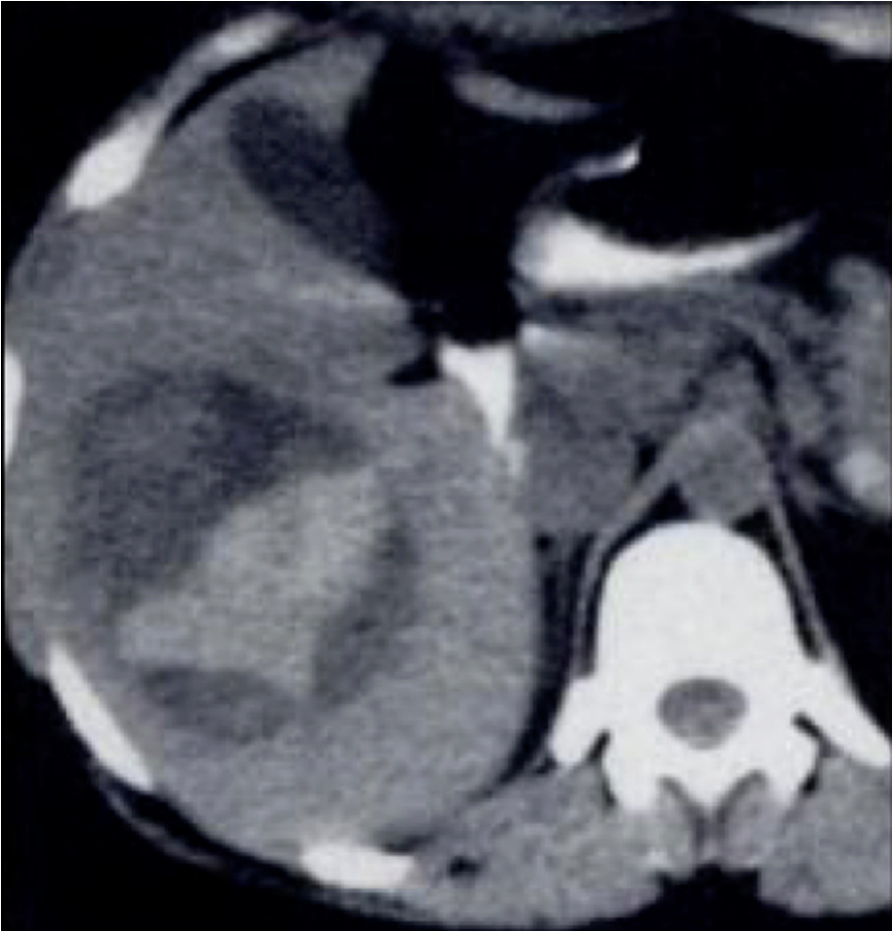
**A 48-year-old man with hepatocellular carcinoma.** Axial unenhanced CT image of the abdomen showing a hepatic mass on the right lobe with hyperattenuating components suggesting acute bleeding.

Intratumoral hemorraghe is suggested by hyperechogenicity at US or hyperattenuating components at unenhanced CT (Figure 12). Hematomas are relatively hyperdense to the unenhanced liver parenchyma during the acute phase (typically 45–70 HU), but the attenuation gradually decreases with time and may be lower in patients with decreased serum hematocrit level. At MRI, foci of high T1 signal intensity are seen in acute hemorrhage, however, the signal intensity of blood is also variable and dependent on the age of hemorrhage [40,61,63].

At unenhanced CT, hemoperitoneum manifests as high attenuation ascites (typically 30–45 HU). Subcapsular hematomas are elliptical high-attenuation collections bound by the organ capsule in which they originate. Thus, on CT images, the highest-attenuation hematoma, or sentinel clot, is that closest to the site of bleeding. Foci of active extravasation of intravenous contrast material extending into or around the hematoma indicate ongoing bleeding. Angiography of the visceral arteries not only helps identify the source of bleeding but also assists in treatment [40,61,63].

## Conclusion

Nowadays, the number of oncological visits is increasing in clinical practice, especially those related to acute and insidious noninfectious oncologic complications. Imaging methods play an essential role in diagnosis and as soon as the support team recognizes these conditions, the most appropriate therapeutic approach may be provided improving the quality of life and survival of these patients.

## Competing interest

The authors or author’s institutions have no competing interest.

## Authors’ contributions

MDG and AGVB drafted the manuscript and provided the presented cases. EM, RC, JLG and VK reviewed the manuscript and the presented cases. All authors read and approved the final manuscript.
